# Surface Chemistry of Lead Halide Perovskite Colloidal Nanocrystals

**DOI:** 10.1021/acs.accounts.3c00174

**Published:** 2023-06-22

**Authors:** Luca De Trizio, Ivan Infante, Liberato Manna

**Affiliations:** † NanoChemistry, Istituto Italiano di Tecnologia, Via Morego 30, 16163 Genova, Italy; ‡ BCMaterials, Basque Center for Materials, Applications, and Nanostructures, UPV/EHU Science Park, Leioa 48940, Spain; § Ikerbasque Basque Foundation for Science, Bilbao 48009, Spain

## Abstract

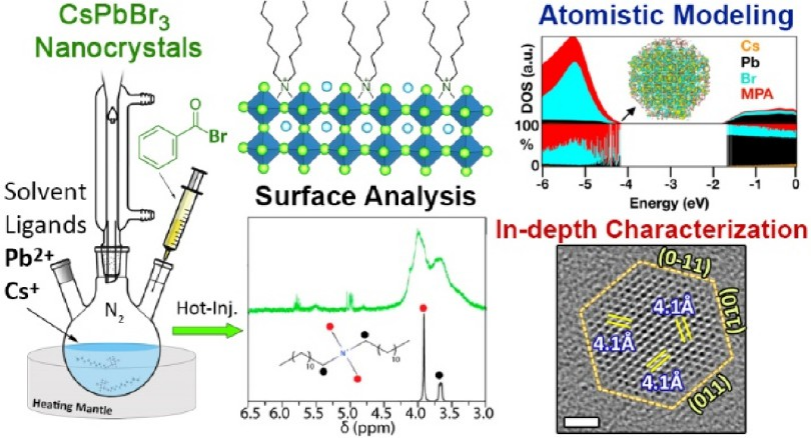

The surface chemistry of lead halide perovskite nanocrystals (NCs) plays a major role in dictating their colloidal and structural stability as well as governing their optical properties. A deep understanding of the nature of the ligand shell, ligand–NC, and ligand–solvent interactions is therefore of utmost importance. Our recent studies have revealed that such knowledge can be harnessed following a multidisciplinary approach comprising chemical, structural, and spectroscopic analyses coupled with atomistic modeling. We show that specific surface terminations can be produced only by employing flexible and versatile syntheses that enable to work under desired conditions. In this Account, we first describe our studies aimed at synthesizing CsPbBr_3_ NCs with various surface terminations. These include CsPbBr_3_ NCs prepared under Br- and oleylamine-rich conditions, which feature a ligand shell composed of alkylammonium-Br species and a photoluminescence quantum yield (PLQY) of ∼90%. On the other hand, taking advantage of the inability of secondary amines to bind to the perovskite NCs surface, we could prepare cuboidal CsPbBr_3_ NCs bearing a Cs-oleate surface termination and a PLQY of 70% by employing oleic acid and secondary alkylamines. In the quest to identify ligands that can bind more strongly than oleates or primary alkylammonium ions to the surface of NCs already in the synthesis step, we used phosphonic acids as the sole ligands in the CsPbBr_3_ NCs synthesis, which yielded NCs with a truncated octahedron shape, high PLQY (∼100%), and a PbBr_2_-terminated surface passivated by hydrogen phosphonates and phosphonic acid anhydride. The surface chemistry and the stability of perovskite NCs were investigated via ad-hoc postsynthesis treatments. We found, for example, that reacting oleylammonium-Br-terminated NCs with stoichiometric amounts of neutral primary alkylamines (or their conjugated acids) led to a partial replacement of oleylammonium ions with new alkylammonium ions (following a deprotonation/protonation mechanism), which resulted in a boost of the PLQY (up to 100%) and of the NCs’ colloidal stability. Similar results in terms of optical properties were achieved by treating Cs-oleate-terminated NCs with alkylammonium-carboxylate or quaternary ammonium-Br (namely, didodecyldimethylammonium-Br, DDA-Br) couples. Interestingly, when the native NCs are ligand exchanged with DDA-Br, the ligand shell is then composed of species not bearing any proton. This, in turn, enabled us to study the interaction of such NCs with a variety of ligands under completely aprotic conditions wherein these DDA-Br-capped NCs were basically inert. The only exceptions were carboxylic, phosphonic, and sulfonic acids that were capable of stripping surface DDA-Br couples. As a note, most studies on CsPbBr_3_ NCs to date have focused primarily on choosing ligands with specific anchoring groups rather than on tuning the length and type of alkyl chains, as this is time-consuming and requires a large number of syntheses. Our recent developments in the computational chemistry of colloidal NCs are expected to provide a pivotal role in this direction since they can be integrated with machine learning models to investigate with greater details the ligand–NC, ligand–ligand, and ligand–solvent interactions and ultimately find optimal candidates through the prediction of surfactant properties using high-throughput data sets.

## Key References

- BodnarchukM. I.; BoehmeS. C.; ten BrinckS.; BernasconiC.; ShynkarenkoY.; KriegF.; WidmerR.; AeschlimannB.; GüntherD.; KovalenkoM. V.; InfanteI.Rationalizing and Controlling the Surface Structure and Electronic Passivation of Cesium Lead Halide Nanocrystals. ACS Energy Lett.2019, 4( (1), ), 63–7410.1021/acsenergylett.8b0166930662955PMC6333230.^1^*The CsPbBr_3_ NC’s surface structure and its effect on the emergence of trap states has been modeled using density functional theory. The typical observation of a degraded luminescence upon aging and the luminescence recovery upon postsynthesis surface treatments are rationalized.*
- ImranM.; CaligiuriV.; WangM.; GoldoniL.; PratoM.; KrahneR.; De TrizioL.; MannaL.Benzoyl Halides as Alternative Precursors for the Colloidal Synthesis of Lead-Based Halide Perovskite Nanocrystals. J. Am. Chem. Soc.2018, 140( (7), ), 2656–266410.1021/jacs.7b1347729378131PMC5908184.^2^*New synthesis approach for lead halide perovskite colloidal NCs based on the use of benzoyl halides as the halide precursors was developed. This synthesis route allows us to independently tune the quantity of both metal cations and halide precursors in the synthesis.*
- ZhangB.; GoldoniL.; ZitoJ.; DangZ.; AlmeidaG.; ZaccariaF.; de WitJ.; InfanteI.; De TrizioL.; MannaL.Alkyl Phosphonic Acids Deliver CsPbBr_3_ Nanocrystals with High Photoluminescence Quantum Yield and Truncated Octahedron Shape. Chem. Mater.2019, 31( (21), ), 9140–914710.1021/acs.chemmater.9b03529.^3^*CsPbBr*_3_*NCs are synthesized with alkylphosphonic acids as the sole ligands. The use of such surfactants yields PbBr*_2_*-terminated NCs (a surface termination hardly observed for these NCs), with a truncated octahedron shape, near-unity PLQY, and stability against dilution.*
- ImranM.; IjazP.; GoldoniL.; MaggioniD.; PetralandaU.; PratoM.; AlmeidaG.; InfanteI.; MannaL.Simultaneous Cationic and Anionic Ligand Exchange For Colloidally Stable CsPbBr_3_ Nanocrystals. ACS Energy Lett.2019, 4( (4), ), 819–82410.1021/acsenergylett.9b00140.^4^*The complete replacement of Cs-carboxylate species from the surface of CsPbBr*_3_*NCs can be attained by employing quaternary alkylammonium-bromide salts. The resulting NCs feature a near-unity PLQY, stability against dilution, and a ligand shell that cannot lose or gain protons.*

## Introduction

**Figure 1:**
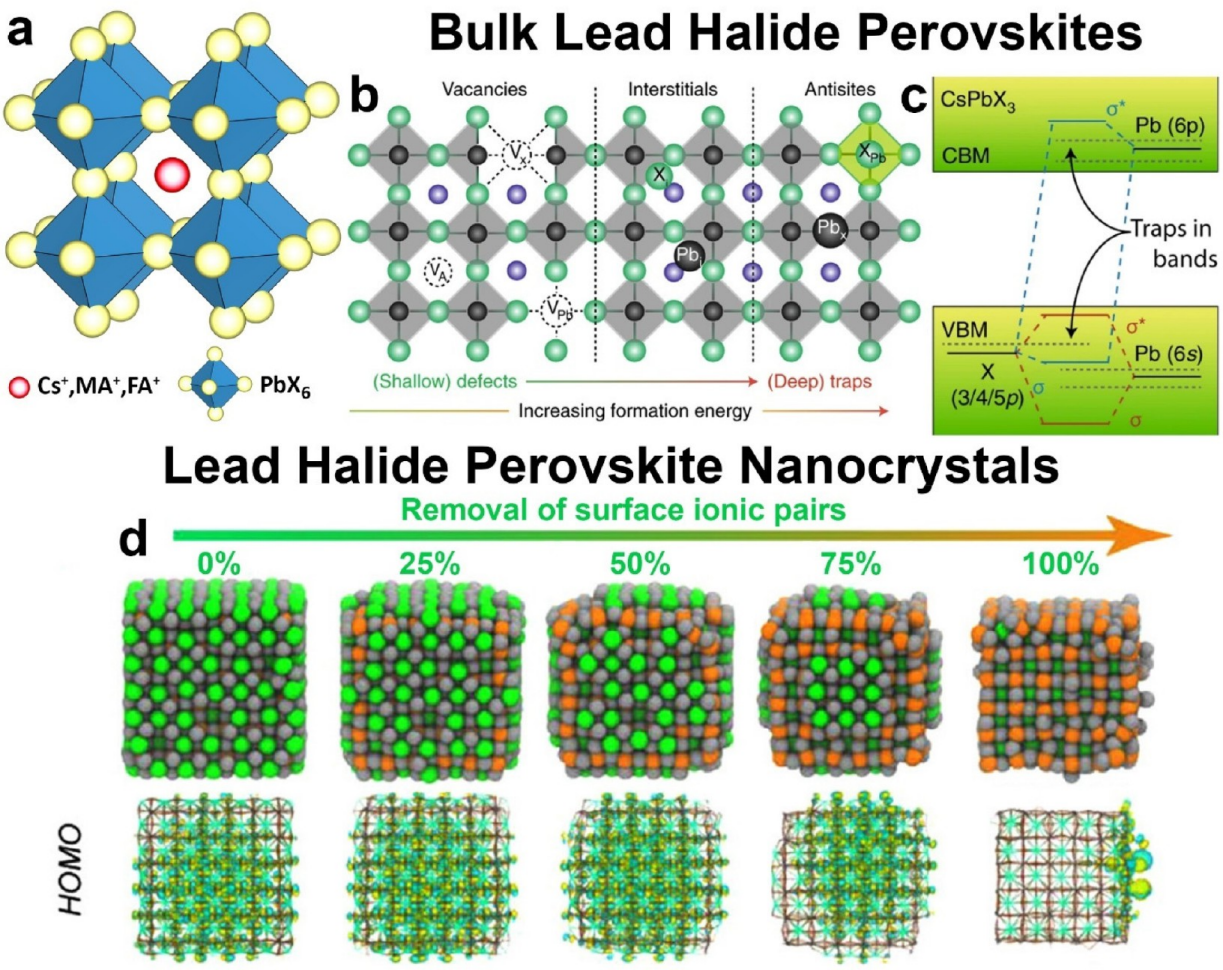
Schematic representation of a) the LHP crystal structure; b) typical defects present in the LHP lattice; c) LHP electronic structure at the band edges; d) variation of the highest occupied molecular orbital (HOMO) composition as a function of extent of removal of surface-bound ion couples. b,c) Reproduced with permission from ref (8). Copyright 2018 Springer Nature. d) Adapted with permission from ref (1). Copyright 2018 American Chemical Society.

Lead halide perovskites (LHPs), with general formula APbX_3_ (where the A^+^ cation stands for Cs^+^, methylammonium -MA, or formamidinium -FA, while X = Cl, Br, I) (Figure 1a), have tunable band gaps and high absorption coefficients that make them attractive in solar energy conversion applications, and at the same time their high carrier mobility makes them good candidates in electronic and optoelectronic devices.^5^ LHPs are also relatively inexpensive to manufacture and can tolerate a high density of defects without significant degradation of their performance. This allows them to be easily processed compared with more traditional semiconductors. In these materials, the most likely structural defects are represented by vacancies (Figure 1b). However, contrary to more traditional semiconductors, localized states originating from these vacancies are either shallow or nested in the valence/conduction bands, hence they are relatively benign (Figure 1c). Nanocrystals (NCs) of lead halide perovskites (LHP) have taken over the scene from “classical” semiconductors, such as those belonging to the II–VI and IV–VI classes, from 2015 onward.^5−7^ This is mainly due to their peculiar optical properties, such as remarkably narrow (<100 meV) and bright photoluminescence (PL) emission that can be easily tuned across the visible range, with quantum yield (QY) approaching near-unity values. In addition, LHP NCs are easily synthesized as colloidal inks, which renders them particularly interesting for optoelectronic applications.^5^

In previous work from some of us, it was found that LHP NCs are markedly different from their bulk counterpart in terms of defect formation energies.^9^ In bulk LHPs, charged and neutral defects are expected to be stabilized inside the lattice and travel through the material extensively before recombining. In contrast, our study on the formation energy of defects in perovskite NCs has shown that they are mostly located at the surface, and even when they are generated in the core of the NC they can easily travel to the surface through vibrational relaxation.^9^ Typical defects on the NC surface are vacancies that are formed by the removal of ionic pairs, such as CsBr or Cs-carboxylate and alkylammonium-Br (see the detailed discussion below) (Figure 1d). These vacancies, if present in small quantities, usually do not affect the electronic structure of a NC (Figure 1d).^9,10^ A previous study by some of us has shown that midgap (trap) states can indeed arise when a considerable number of ion pairs are removed from the NC surface, leaving behind highly undercoordinated halide ions (Figure 1d).^1^ Hence, although most of the reported colloidal routes deliver LHP NCs with high PLQY values, such NCs must be handled with care to avoid the loss of too many surface ion pairs and the consequent formation of trap states, with the worsening of PLQY and colloidal stability. The conditions for the detachment of ion couples can be met either during the synthesis or, more likely, during the postsynthetic treatment of the NCs, for example, by the common washing procedures with various solvents. As such, in LHPs NCs, as in classical semiconductor NCs, understanding the interactions of the inorganic cores with their surrounding organic medium is essential to better control not only their nucleation and growth, but also their optical properties and integration in devices.^5^

This Account summarizes our current understanding of the surface chemistry of LHP NCs and our contribution to this field. It should be emphasized that, in some aspects, the characterization of the LHP NCs’ surface has turned out to be more challenging than that of “classical” semiconductor NCs, mainly due to the ease by which LHP NCs lose colloidal stability and/or structural integrity when handled. This is mainly ascribed to the ionic bonding that characterizes both the LHP NCs’ core (structural lability) and the ligand–surface interactions (colloidal lability). To better describe these points and to elucidate the surface chemistry of LHP NC materials, we take as a case study CsPbBr_3_ NCs in light of several key aspects: (i) for analytical reasons, the absence of ammonium species in the NCs’ core enables a precise characterization of the ligand shell when alkylammonium ions are used as surfactants; (ii) all-inorganic LHPs are less labile than the corresponding organic–inorganic counterparts;^11^ and (iii) from a colloidal synthesis point of view, all-inorganic LHP NCs are easier to prepare (most likely because MA- and FA-based precursors decompose at low temperatures).^2^

### CsPbBr_3_ Nanocrystal Model and Ligand Classification

**Scheme 1:**
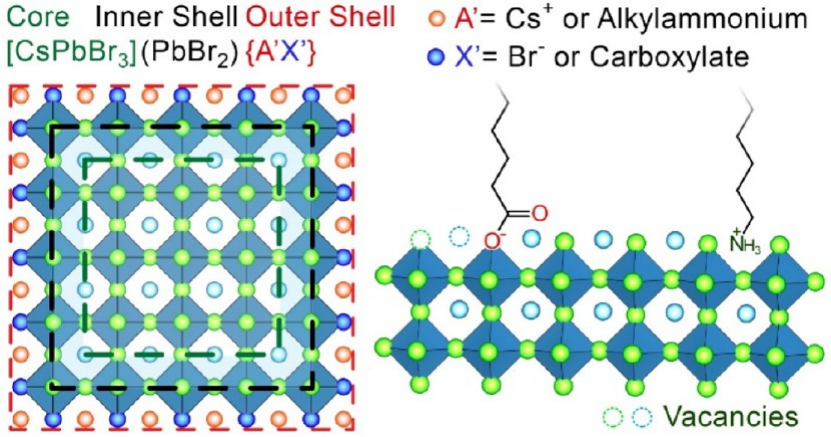
Schematic Representation of a CsPbBr_3_ NC and Its Surface Termination in Which Ligands Occupy Lattice Sites

CsPbBr_3_ NCs can be described as objects made of a stoichiometric CsPbBr_3_ core, a PbBr_2_ “inner shell”, and an A′X′ outer shell [CsPbBr_3_](PbBr_2_){A′X′}(Scheme 1).^1^ The outer shell includes A′ cations, which can be either Cs^+^ or alkylammonium ions, and X′ anions, comprising Br^–^ and alkyl carboxylate anions. This model takes into account several important aspects characterizing CsPbBr_3_ NCs: (i) the most common ligands employed in their synthesis are primary alkylamines and carboxylic acids, which bind to the surface in their protonated/deprotonated form;^11^ (ii) the A′X′ termination is the most frequently observed one, as these ligands bind to the (001), (100), and (010) facets, thus delivering cuboidal NCs.^8^ Only in rare cases has CsPbBr_3_ been shown to be PbBr_2_-terminated (for example, when passivated with alkyl phosphonic acids, see below).

st Following these points and with the benefit of hindsight, we argue that the covalent bond classification, which sorts surfactants as X-, Z-, and L-type depending on the electrons shared in bonding to NCs surface sites^12^ and which is typically employed to describe the ligand–NC interaction in the case of “classical” semiconductors, should be avoided in the case of LHP NCs as inappropriate.^10,13−16^ A more realistic picture can be attained with the charge-orbital balance model, in which NCs are considered to be neutrally charged and all of the species involved are in their thermodynamically most favorable oxidation state (e.g., +1 for Cs and alkylammonium, +2 for Pb, and −1 for Br and carboxylates).^10,17,18^ Indeed, LHP NCs are characterized by highly ionic character and ligands are bound to the surface as ions occupying surface sites (e.g., alkylammonium substituting for Cs^+^ cations and carboxylates replacing Br^–^ anions, Scheme 1),^19,20^ therefore in a noncovalent way. Interestingly, while in “classical” semiconductor NCs charged ligands can in some cases induce doping (e.g., I^–^ ions on the surface of a PbS NC could form gaseous I_2_ and thus act as a p-type dopant), in LHP NCs doping induced by ligands has never been proven, strengthening the conclusion that in such systems the ligands bind as overall neutral ion pairs, thus ensuring that the whole system is charge-balanced (i.e., intrinsic behavior).^17^

### Control Over the Surface Termination of CsPbBr_3_ NCs via Direct Synthesis

**Figure 2:**
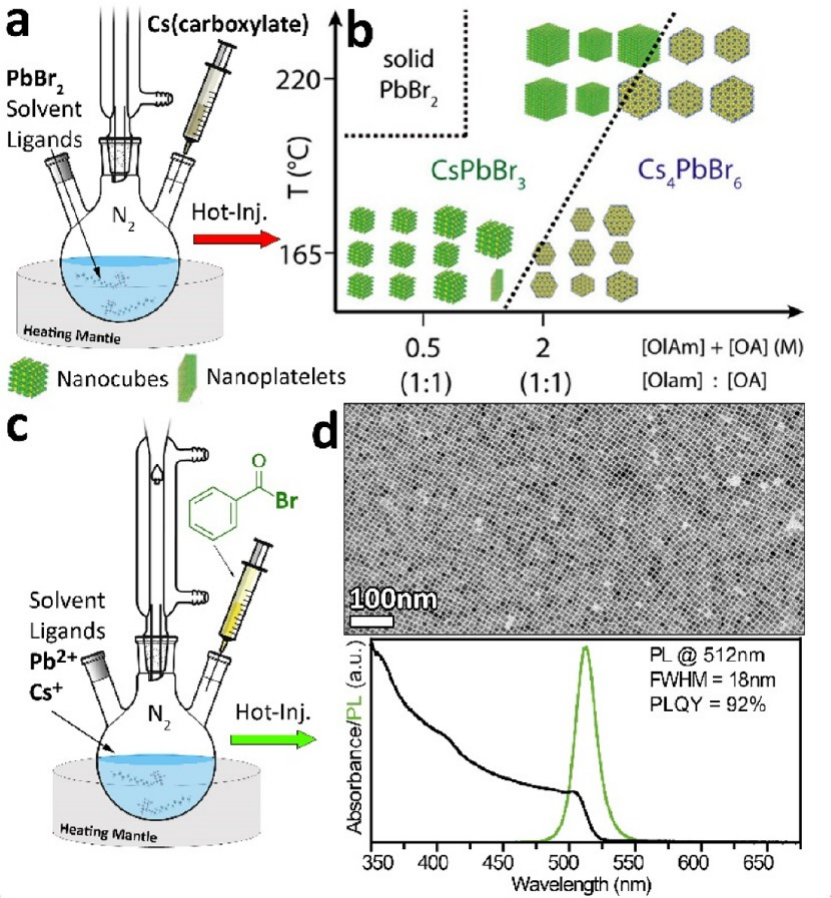
Schematic representation of a) the classical hot-injection approach and c) the one relying on benzoyl-bromide. b) Range of products that can be observed with the classical approach when varying the relative amounts of Olam and OA and the reaction temperature. Reproduced with permission from ref (21). Copyright 2018 American Chemical Society. d) TEM picture and optical properties of CsPbBr_3_ NCs synthesized with the benzoyl-halide approach under Br- and Olam-rich conditions. Reproduced with permission from ref (2). Copyright 2018 American Chemical Society.

As highlighted above, mastering the surface chemistry of LHP NCs is of utmost importance to define and tune their colloidal and structural stability and, ultimately, their optical properties. From an experimental standpoint, control over the surface of LHP NCs is made possible by the flexibility of their colloidal synthesis procedure. In this context, the first hot-injection routes devised for the synthesis of CsPbBr_3_ NCs, dating back to 2015,^5^ relied on metal halide salts (e.g., PbX_2_) as both the metal cation and halide precursors (Figure 2a). This entailed two main limitations: (i) the halide/metal cations ratio was fixed, with Br being inevitably deficient, and (ii) the metal halide salts had to be dissolved in proper high-boiling solvents or ligands.^5,21,22^ Regarding the first limitation, a Br-poor environment typically leads to NCs with a Br-poor surface, which, in practice, corresponds to CsBr (or alkylammonium-Br) vacancies and therefore low PLQYs (see the discussion above and Table 1.) This explains the various reports of low PL efficiencies encountered in systems made under Br-poor conditions, independently of the types of ligands employed.^2,5,23^ The main strategy to circumvent such an issue is the use of extra Br sources, for example, metal halide salts, such as ZnBr_2_, bearing metal cations that are not incorporated into the CsPbBr_3_ perovskite lattice.^23,24^ Regarding the second limitation, the dissolution of PbBr_2_ has been achieved with a coordinating agent such as trioctylphosphine oxide (TOPO)^11,22^ or more often by the combined use of an oleylamine (Olam) and oleic acid (OA) (in nonpolar solvents). However, the concentrations and relative ratios of these molecules are critical, as they impose constraints on the temperature at which the synthesis can be carried out (in the case of Olam/OA mixtures, PbBr_2_ can precipitate above 190 °C), and they can also lead to the formation of undesired shapes or phases (Figure 2b). For example, high concentrations of primary alkylamines can favor the growth of CsPbBr_3_ nanoplatelets (as alkylammonium ions start competing with Cs^+^ ions for addition to the growing NCs) or, by heavily complexing PbBr_2_, they can lead to the formation of the “Pb-poor” Cs_4_PbBr_6_ phase.^19−21,25−28^

**Table 1:** Main Features of LHP NCs Obtained via Either the First Hot-Injection Approaches or the Benzoyl-bromide Route

				**Stability**		
**Synthesis type**	**Ligands employed**	**Ligand shell**	**PLQY**	**Colloidal**	**Dilution**	**Specific marks**
hot injection	Olam + OA	oleylammonium-Br + Cs-oleate	60–70%	good	no	Br-poor surface
benzoyl-bromide	mostly Olam	oleylammonium-Br	∼90%	poor	no	Cs-poor, Br-rich surface
	OA + secondary amines	Cs-oleate	∼70%	good	no	AX surface vacancies
	Pas	Pb^2+^-PA^–^, PA^2–^, PA_anhy_^–^	100%	good	yes	trucated octahedron shape; Pb-rich surface

The need to circumvent such limitations, explore a wider range of conditions, and work under a broader range of precursors ratios motivated us in 2018 to devise a new hot-injection protocol based on the use of benzoyl halides as precursors for halides.^2^ They can be injected in a reaction mixture composed of the desired metal cation precursors (e.g., acetates, oxides, and carbonates) and surfactants, triggering the immediate formation of the NCs (Figure 2c). The first protocol that we explored delivered cubic CsPbBr_3_ NCs obtained under Br- and Olam-rich and Cs-poor conditions, characterized by oleylammonium-Br surface passivation (Table 1).^2,18,20^ Indeed, the NCs featured Cs-poor and Br-rich compositions, with a large number of Cs^+^ surface sites being occupied by oleylammonium ions. Such surface termination was found to lead to high PLQY values (∼90%) resulting from an optimal passivation of the surface A′X′ sites (Figure 2d). It should be emphasized that with previous synthesis protocols the achievement of cubic NCs with such surface termination was not straightforward: the restrictions of having to use carboxylic acids and alkylamines in specific ratios typically led to NCs with a mixed Cs-carboxylate and alkylammonium-Br termination (Table 1).^27−29^ In this context, it is notable that different ligand shell compositions have been reported for CsPbBr_3_ NCs made with similar reaction protocols involving mixtures of carboxylic acids and alkylamines. Such apparent discrepancies can be rationalized by considering that 1) cleaning procedures either lead to a partial detachment of ligands or they are not entirely efficient; that is, the samples can still be contaminated by excess free ligands; and 2) it is hard to differentiate bound alkylamines and carboxylic acids via NMR, as both species feature protons in the position α to the amino or carbonyl groups at similar resonances.^15,19,27,28,30^ Also, such resonances can shift depending on the acidity of the medium, plus they are difficult to resolve when these molecules are bound due to signal broadening.^21,30^

**Figure 3:**
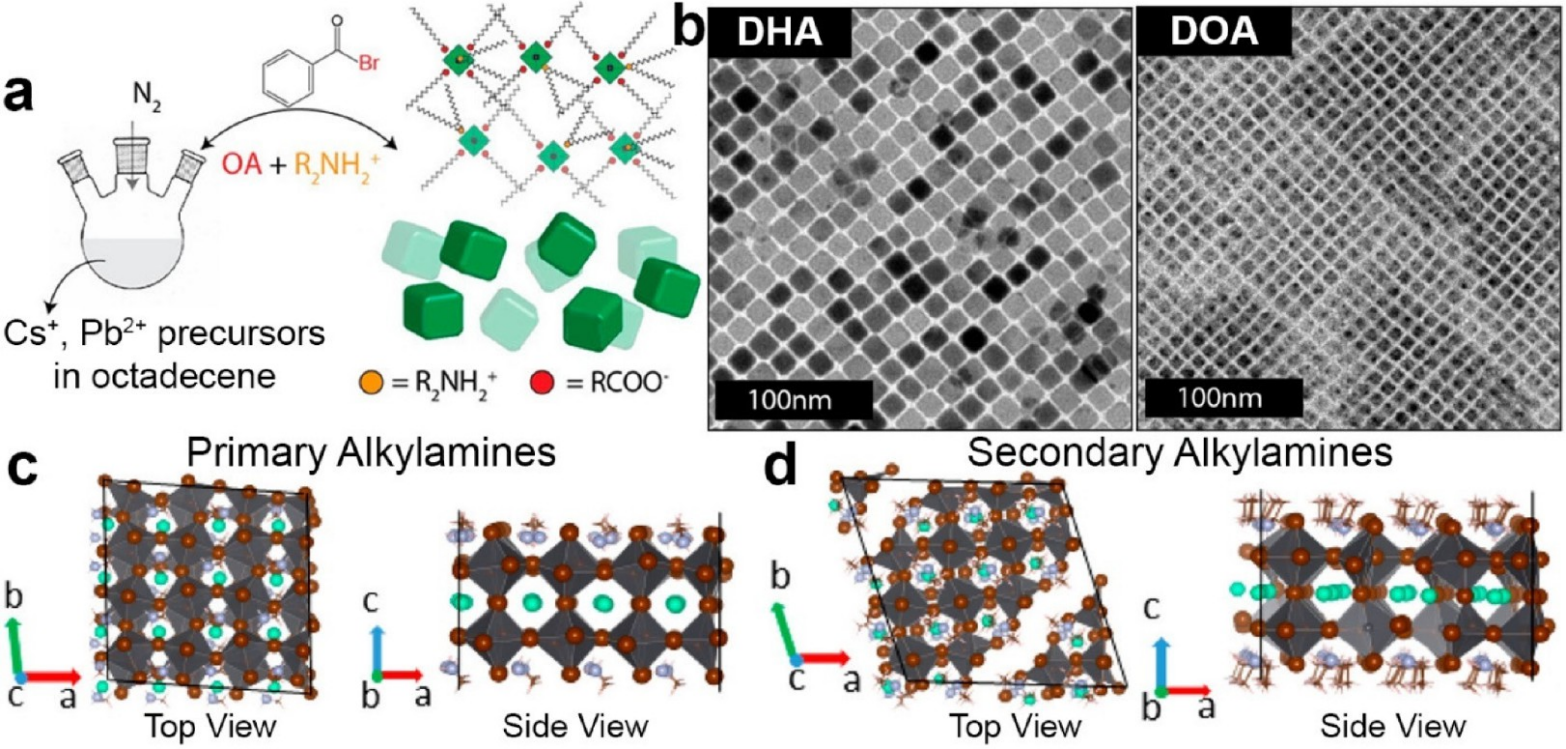
a) Synthesis scheme of CsPbBr_3_ NCs employing secondary alkylamines. b) TEM images of CsPbBr_3_ NCs prepared by employing either dihexylamine (DHA) or dioctadecylamine (DOA). Side and planar views of the binding of c) primary and d) secondary amines in the CsPbBr_3_ lattice. The structural relaxation was carried out at the DFT level of theory. In the case of secondary amines, the anchoring groups are closer to each other than those in primary amines, indicating a larger steric hindrance. Adapted with permission from ref (20). Copyright 2018 American Chemical Society.

Benefiting from the newly established synthesis approach, our next step was to explore a series of surfactants, including secondary amines and phosphonic acids, whose use was previously precluded, as they cannot solubilize PbBr_2_. Using our benzoyl-halide approach, we thus tested secondary amines of variable chain lengths to synthesize CsPbBr_3_ NCs in accordance with OA (Figure 3a).^20^ The main outcome of our study was the discovery that protonated dialkylamines (independent of their chain length) are not able to bind to the surface of LHP NCs. This was rationalized by our density functional theory (DFT) models which indicated that protonated secondary alkylamines, different from primary amines, do not fit well into Cs^+^ surface sites (Figure 3c,d): secondary alkylamines, in order to bind stably to the surface, would need to force their alkyl chains into highly strained geometrical configurations, which in turn would need to locally deform the underlying CsPbBr_3_ lattice. This has several significant implications: i) CsPbBr_3_ NCs made with secondary amines featured a Cs-carboxylate surface termination (Table 1). Our NMR analysis revealed that over 90% of bound surfactants were oleates, and the NCs had a PLQY of ∼60–70%, in agreement with previous reports on analogous surface-terminated NCs.^22^ ii) The lack of competition between Cs^+^ and dialkylammonium ions for binding surface sites led to cuboidal NCs (i.e., no nanoplatelets were observed) regardless of specific reaction conditions (Figure 3b).

**Figure 4:**
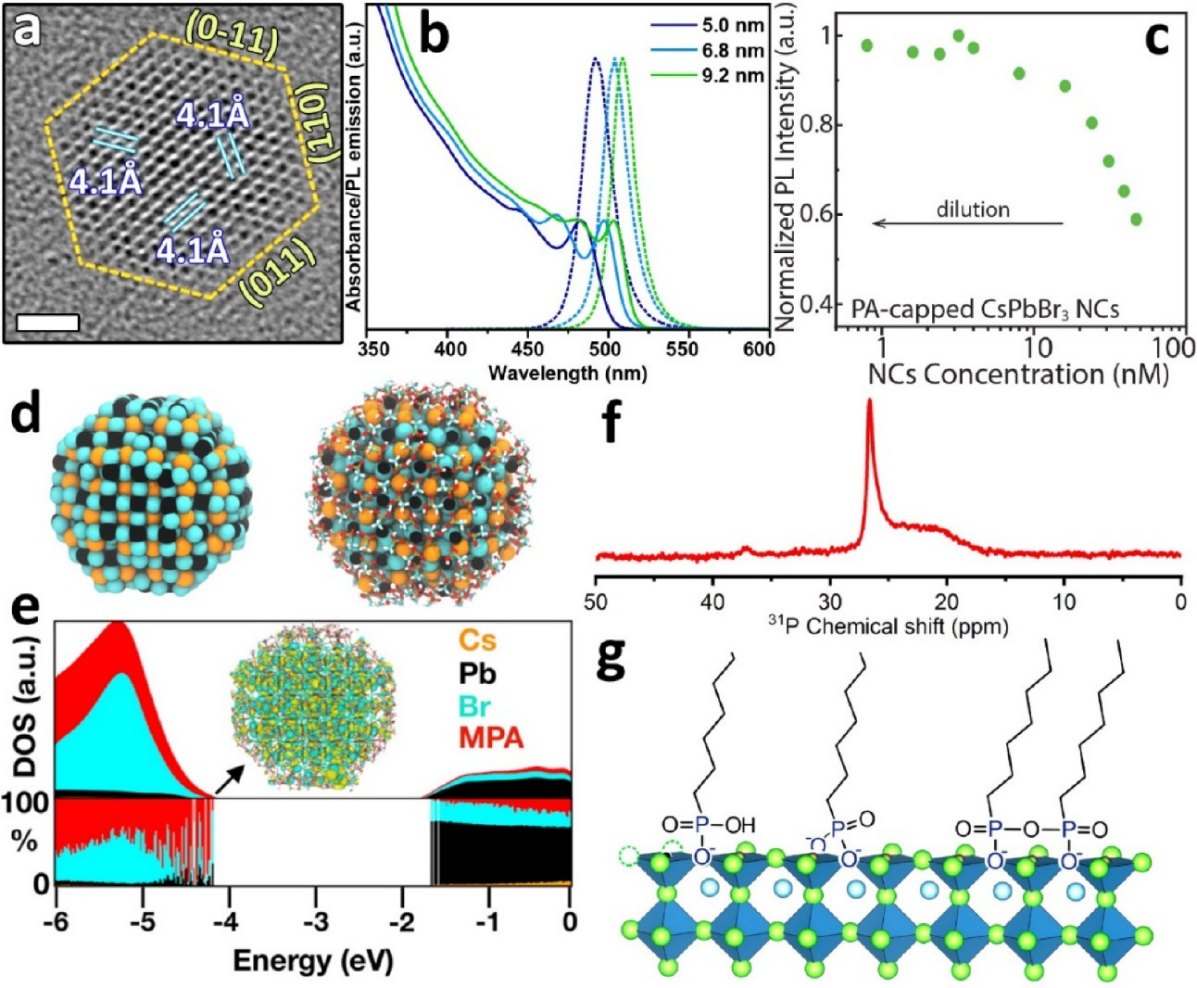
a) High-resolution TEM image of a PA-based CsPbBr_3_ NC showing a truncated octahedron shape. b) Absorbance and PL emission of CsPbBr_3_ NCs of different sizes made with oleylphosphonic acid. c) PL intensity variation as a function of PA-based NCs concentration in toluene. d) Relaxed CsPbBr_3_ NC model of 3.0 nm diameter computed at the DFT level of theory in which methylphosphonate (MPA) ligands have been visually excluded (left) or included (right). (e) Projected density of states (PDOS) on each atom and ligand type of the NC model. A delocalized and trap-free molecular orbital plot of the HOMO state is also shown. f) ^31^P NMR spectrum of PA-based LHP NCs in toluene. g) Schematic representation of the surface termination of PA-based NCs. a,c–e) Reprinted with permission from ref (3). Copyright 2019 American Chemical Society. b,f) Reprinted with permission from ref (32). Copyright 2020 American Chemical Society.

The issue with primary ammonium ions and carboxylate ions is that they are dynamically bound to the NCs’ surface and they can easily gain/lose protons, thus becoming electrically neutral and eventually detaching from the surface, as originally highlighted by De Roo et al.^15,18^ For instance, 90% of NCs are typically lost in the conventional OA/Olam synthesis due to the detachment of ligands, leading to NCs’ aggregation and deterioration of optical properties (drop in PLQY).^13,31^ In the quest for ligands that would be bound more strongly to the surface of NCs, we explored for the first time alkylphosphonic acids (PAs) of different chain lengths (ranging from 1 to 18 carbon atoms) as the sole surfactants in the synthesis of CsPbBr_3_ NCs (Table 1).^3,32^ The resulting NCs had a size that could be easily tuned, ∼100% PLQY and a truncated octahedron shape arising from the presence of (110) and (111) facets in addition to the more typical (001), (100), and (010) facets (Figure 4a–d). The NCs featured a Pb-rich and a Br-poor surface in which alkyl phosphonates bind to the surface (replacing Br^–^ anions) in the form of hydrogen phosphonate (PA^1–^), phosphonate (PA^2–^), and phosphonic acid anhydride (PA_anhy_^1–^) (as revealed by NMR analyses) (Figure 4f,g). NMR analysis also indicated that when working at high temperatures (i.e., 160 °C) PA_anhy_^1–^ species were preferentially passivating the NCs’ surface, while at lower temperatures (i.e., 100 °C) PA^–^ and especially PA^2–^ moieties dominated the ligand shell. Such ligands, as indicated by our DFT calculations, not only stabilize Pb-rich surfaces (yielding NCs with a clean band gap, Figure 4d,e) more favorably than Cs-rich ones, but also have similar binding affinities for the (001) and (110) facets, thus explaining the observed truncated octahedron shape. Also, when highly diluted in a solvent such as toluene (a condition that may promote partial ligand detachment and a drop in PLQY) these NCs preserved their high PLQY, differently from the NCs coated by ligand pairs such as oleylammonium-Br or Cs-oleate, for which the PLQY dropped instead (Table 1). This simple test indicates that these phosphonic acid-derived ligands are more tightly bound to the surface of the NCs.

### Post-Synthesis Treatments of CsPbBr_3_ NCs

#### Exposure to Exogenous Molecules/Ligands

As anticipated above, CsPbBr_3_ NCs synthesized with alkylamines and/or carboxylic acids are known to be difficult to handle since they can easily lose ligands and thus aggregate. This surface lability, on the other hand, also represents an opportunity for chemists to study in more detail the surface of NCs, as this means that the surface is readily accessible to exogenous molecules. To better understand the behavior of perovskite NCs when exposed to different molecules, we performed a systematic study in which CsPbBr_3_ NCs, terminated with two types of ligand pairs (either Cs-oleate or oleylammonium-Br) were treated with either primary alkylamines or their conjugated acids.^18^ The study is summarized below.

##### Oleylammonium-Br-Terminated CsPbBr_3_

**Figure 5:**
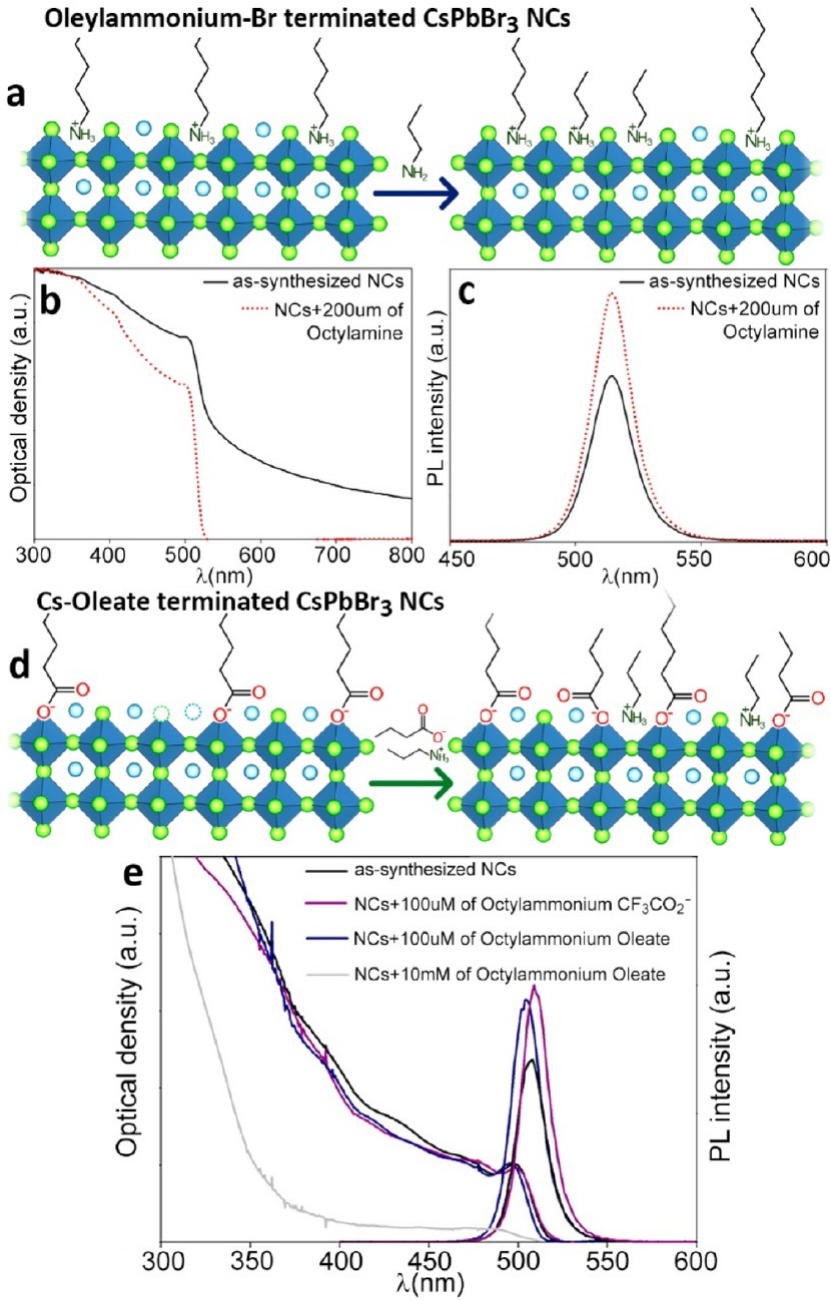
a) Schematic representation of the ligand exchange between oleylammonium-Br CsPbBr_3_ NCs and octylamine. b) Absorption and c) PL emission variation upon the addition of stoichiometric amounts of octylamine. d) Schematic representation of the ligand exchange between Cs-oleate CsPbBr_3_ NCs and octylammonium-carboxylates salts. e) Absorption and PL emission variation upon the addition of different salts. Adapted with permission from ref (18). Copyright 2019 American Chemical Society.

Oleylammonium-Br CsPbBr_3_ NCs, featuring ∼90%PLQY and nonoptimal colloidal stability (the NCs partly aggregate, resulting in opaque solutions that scatter visible light), were exposed to stoichiometric amounts of octylamine (Table 2). This treatment increased their colloidal stability with no apparent modification of their PLQY and composition (Figure 5a–c). By NMR analysis, we confirmed that added short-chain amines undergo protonation (most likely by capturing protons from bound oleylammonium species) and bind to the NCs’ surface, where they partially replace the bound oleylammonium ions. This reaction overall results in a mixed alkylammonium ligand shell, known to strongly enhance the colloidal NCs’ stability (Figure 5a).^33,34^ Similar results were observed when exposing the oleylammonium-Br CsPbBr_3_ NCs to the conjugate acids of octylamine (e.g., octylammonium trifluoroacetate/oleate), most likely for the same reasons (i.e., replacement of oleylammonium with octylammonium ions) (Table 2). Upon further increase of octylamine addition, a reshaping of the NCs was observed, followed by etching and phase change (i.e., formation of the PbBr_2_-depleted Cs_4_PbBr_6_ phase). Such results, in agreement with other works/reports,^35−37^ indicated that an excess of added amines is able to extract PbBr_2_ species from the NCs and promote their transformation.

**Table 2:** Main Features of LHP NCs Obtained via Post-Synthesis Ligand Exchange Procedures^a^

				**Stability**		
**Starting NCs**	**Exogenous molecule**	**Ending NCs**	**PLQY**	**Colloidal**	**Dilution**	**Specific marks**
Olam-Br	octylamine	Olam-Br	100%	good	no	works only under stoichiometric amounts
		octa-OA				
Olam-Br	octa-OA	Olam-Br	100%	good	no	works only under stoichiometric amounts
		octa-OA				
Cs-OA	octa-OA	Cs-OA	100%	good	no	octa-OA fills available surface AX vacancies
		octa-OA				
Cs-OA	DDA-Br	DDA-Br	100%	good	yes	absence of protons on NCs surface
DDA-Br	oleic acid, olpa	DDA-Br (partial stripping)	100%	good	yes	aprotic conditions; up to 40% of DDA-Br stripped

a Olam-Br = oleylammonium-Br; Cs-OA = Cs-oleate; octa-OA = octylammonium-oleate; octa-Br = octylammonium-Br; and olpa = oleylphosphinic acid.

##### Cs-Oleate Terminated CsPbBr_3_

Cs-Oleate CsPbBr_3_ NCs, characterized by nonoptimal PLQY (∼70% max) and good colloidal stability, featured different behavior when exposed to amines/ammonium ions: (i) the addition of stoichiometric amounts of octylamine led to etching of the NCs; (ii) adding octylammonium-carboxylates (i.e., oleate or trifluoroacetate) in stoichiometric amounts led to a significant enhancement of the PLQY (exceeding 90%) without altering the morphology of the NCs; and (iii) large amounts of amine/ammonium species induced morphological and structural transformations (Figure 5d,e). These results suggest that neutral amines are not able to gain protons and therefore do not bind the NCs’ surface but only displace surface PbBr_2_ (and/or Cs-oleate ion pairs). On the other hand, ammonium-carboxylates, as indicated also by NMR analyses, can replace part of the Cs-oleate couples and likely also fill available surface AX vacancies, hence boosting the NCs PLQY (Figure 5d and Table 2).

In summary, our works on oleylammonium-Br- and Cs-oleate-terminated NCs evidenced not only that CsPbBr_3_ NCs exhibit a reactivity that depends on their surface termination, but also that postsynthesis treatments must be performed with well-defined amounts of exogenous molecules. This is due to the existence of a delicate equilibrium between the formation of ligand–NC bonds (replacement/addition of surface ion couples) and the solubilization of the NC cores, as a consequence of the ionic bonding and low lattice energy (structural lability) characterizing these systems.

##### Quaternary Ammonium-Br CsPbBr_3_ Termination

**Figure 6:**
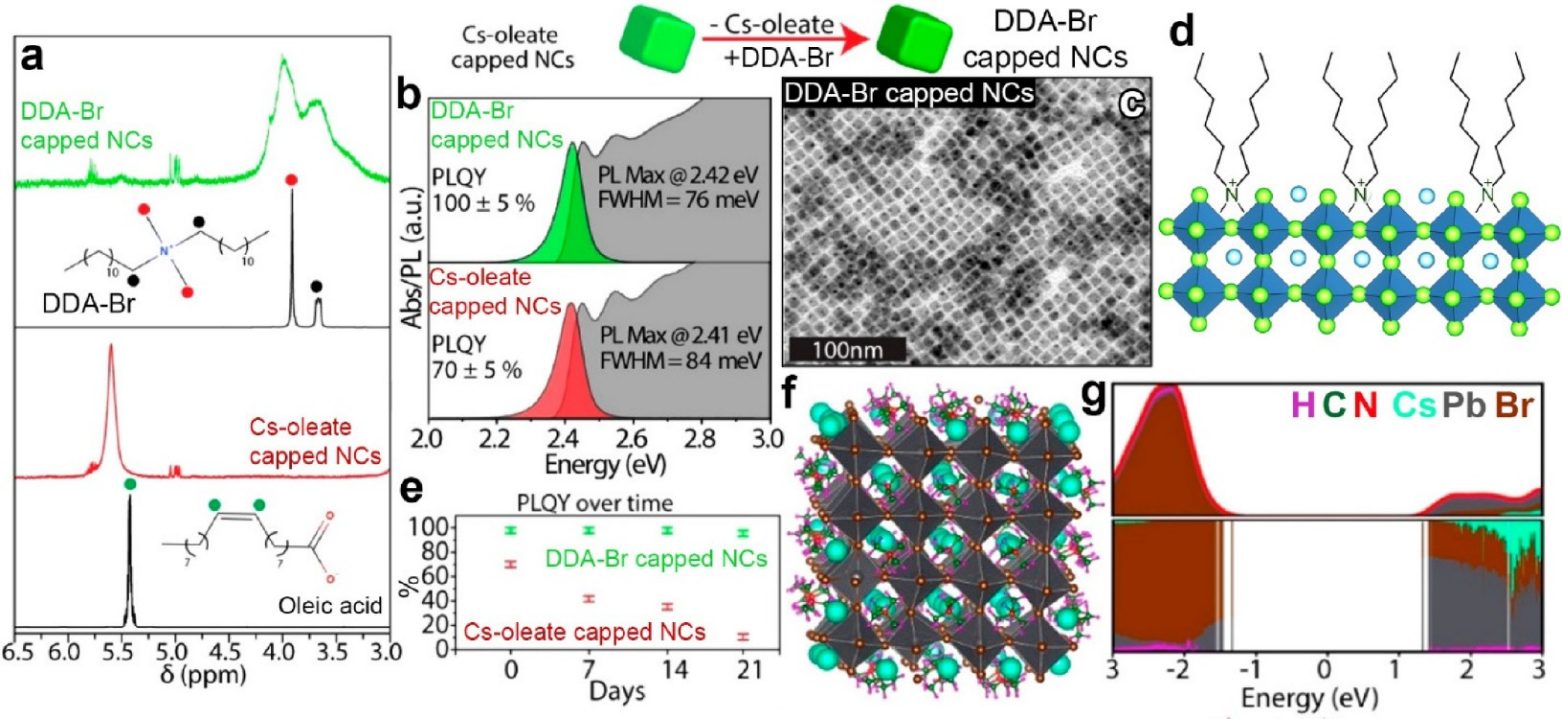
Ligand exchange between Cs-oleate CsPbBr_3_ NCs and DDA-Br: a) ^1^H NMR spectra before and after the exchange; b) absorption and PL emission of starting and product NCs; c) TEM picture of DDA-Br capped NCs; d) schematic representation of the resulting surface termination; e) stability of the nanocrystals upon storage in air; f) relaxed structure of a 2.4 nm CsPbBr_3_ NC model passivated with tetramethylammonium-bromide computed at the DFT level of theory; and g) PDOS on each atom and ligand type. The band gap is free of traps. Adapted with permission from ref (4). Copyright 2019 American Chemical Society.

Building upon the above findings, we shifted our focus toward implementing a surface passivation scheme that eliminates the involvement of protons and restricts the ability of bound species to gain or lose protons. To do so, we devised a ligand exchange procedure to replace surface Cs-oleate couples in CsPbBr_3_ NCs with quaternary alkylammonium-Br, namely, didodecyldimethylammonium-Br (DDA-Br) (Table 2).^4^ We found out that such an exchange leads not only to a complete replacement of Cs-oleate couples with DDA-Br ones, as emerging in our NMR studies (Figure 6a,d), but also to an apparently unbalanced replacement of 0.1 Cs^+^ with 0.4 ammonium ion per each Pb in the NCs, as revealed by elemental analyses. These findings indicated that the initial Cs-oleate-capped NCs did not present a completely passivated outer shell in terms of filling AX sites. Following the exchange, the NCs acquired a near-unity PLQY and excellent colloidal stability (Figure 6b,c,e). The latter result, combined with the observations from our previous study (where we treated Cs-oleate-capped NCs with octylammonium carboxylates) and with DFT calculations, shed some light on the poor PLQY values commonly observed for Cs-carboxylate CsPbBr_3_ NCs, which we summarize here. Carboxylates can efficiently passivate surface Br vacancies, hence Cs-carboxylate-terminated NCs can potentially feature a near-unity PLQY.^34^ Therefore, we attribute the low PL efficiencies of such NCs to a significant presence of AX surface vacancies. These vacancies form as a consequence of the reaction conditions under which Cs-carboxylate CsPbBr_3_ NCs have been synthesized, namely, Cs-poor or Br-poor ones.^20,22^ However, such vacancies can be easily filled by alkylammonium-carboxylate or DDA-Br couples, resulting in highly luminescent NC systems.

Indeed, our DFT calculations indicated that quaternary ammonium ions, different from secondary ammonium ions, are able to fit into Cs^+^ surface sites, leading to a clean band gap (Figure 6f,g), which explains the effectiveness of the ligand exchange procedure. Interestingly, the presence of DDA^+^ on the NCs’ surface led to their stability upon dilution in toluene, similar to what we observed with PA-capped NCs (Figure 4c), while Cs-oleate and oleylammonium-Br NCs are well known to precipitate at high dilutions due to ligand detachment.^3,15,31^ This phenomenon is a common issue affecting perovskite NCs for which the solubility of ligands competes with their coordination to the surface.^13,14,34^ The stability against dilution has been generally ascribed to the binding strength of the surfactant: a strongly bound species is less favorably detached from the surface.^14^ On the other hand, our calculations revealed that all of the systems under analysis feature similar binding energies: ∼50–60 kcal/mol for PA-Pb^2+^, 51.3 kcal/mol for Cs-oleate, 45.3 kcal/mol for primary alkylammonium-Br, and 48.2 kcal/mol for DDA-Br.^3,4,22,32^ Overall, these data strongly indicate that the stability against dilution or cleaning with polar/apolar solvents cannot be ascribed to the binding affinity of the ligands but most likely to ligand–ligand and ligand–solvent interactions (i.e., a lower solubility of DDA-Br or Pb-phosphonates in toluene with respect to Cs-oleate or oleylammonium-Br limits the detachment of the former ligand couples upon dilution).

#### Aprotic Environment

**Scheme 2:**
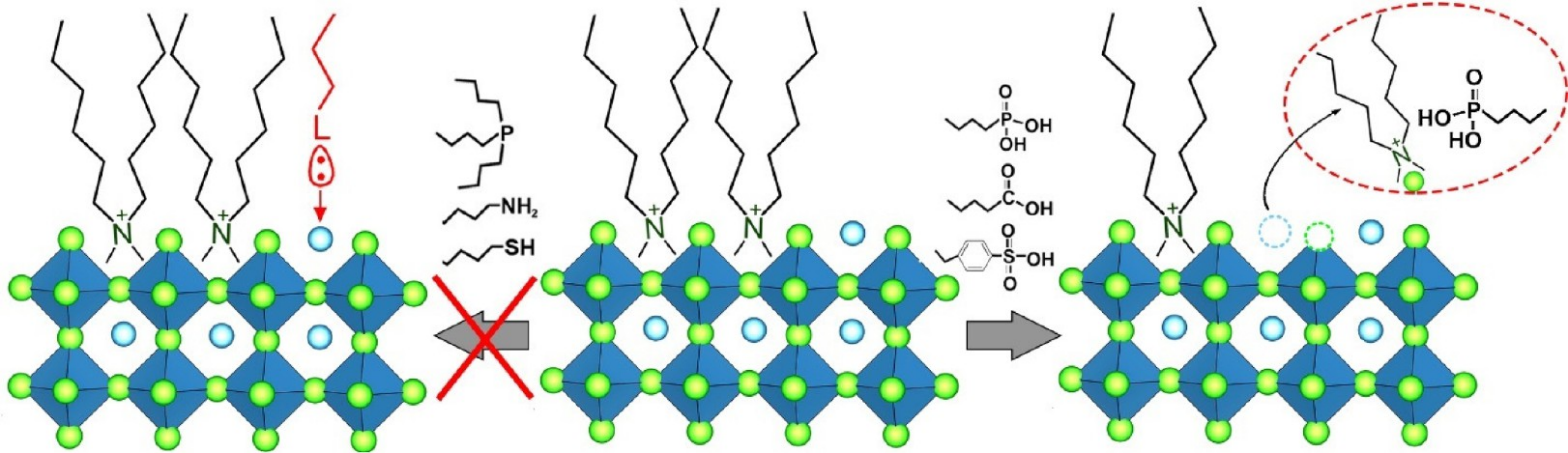
Exposure of DDA-Br NCs to Exogenous Molecules under Completely Aprotic Conditions

DDA-Br NCs represent the first case of a perovskite system in which protons are completely absent and cannot interact directly with bound ligands (no possibility to protonate/deprotonate DDA^+^). For this reason, we studied the interaction between DDA-Br-capped CsPbBr_3_ NCs with a wide range of exogenous molecules, in a completely aprotic environment, at both the experimental and DFT levels.^16^ The exogenous species were added to NC dispersions in toluene in different amounts, ranging from 1 to 10 equiv (large excess) with respect to surface Br sites. We observed that most exogenous species, including thiols, amines (primary, secondary, and tertiary ones), and phosphines, did not interact with DDA-Br NCs; that is, they did not bind as neutral (L-type) ligands or cause any detachment of the native ligands or etching, degradation, or phase transformations (Scheme 2). This result is very surprising considering that neutral primary alkylamines were commonly observed to extract PbBr_2_ species, causing etching/degradation and phase transformation (to the Cs_4_PbBr_6_ phase) of “standard” (e.g., alkylammonium-Br- or Cs-carboxylate-coated) perovskite NCs (see above).^5,18^ Such evidence indicates that the presence of protons, either in “non-well-washed” CsPbBr_3_ NC dispersions or in bound ligand species, catalyzes the etching/phase transformation of these NC systems upon amine addition. On the other hand, organic acids, namely, oleic acid, oleylphosphonic acid, and dodecylbenzenesulfonic acid, were found to etch the NCs, with the degree of etching that depends on the acidity of the exogenous acid (Scheme 2 and Table 2). Experimentally, we observed that oleic acid (the weakest acid in our study) removed only a minor fraction of DDA-Br species, oleylphosphonic acid (a moderate acid) stripped up to 40% of DDA-Br, and dodecylbenzenesulfonic acid (the strongest acid) completely dismantled the NCs.

Interestingly, upon stripping of the DDA-Br moieties, the NCs did not undergo any morphological or optical alteration, thus retaining their unity PLQY values. These results indicate that, when acids are not able to donate their protons, they cannot bind to the surface of the NCs.^29^ They are, however, able to interact in their neutral form with DDA-Br species and eventually provide a useful strategy to control the surface ligand coverage of perovskite NCs. This last achievement is of particular relevance if one considers that in traditional semiconductor NCs the stripping of ligands can be performed in many ways and typically consists of replacing organic ligands with inorganic species, a procedure that generally does not alter the NC core.^38,39^ Instead, in perovskite NCs the extraction of surface ion couples is strongly correlated to the extraction of NCs’ ionic building units, and this can lead to severe reshaping or even dismantling of the NCs.

### Outlook

The studies presented in this Account indicate how the surface of halide perovskite NCs is significantly different from that of “classical” semiconductors, and several key points remain to be assessed more thoroughly. A case in point is the exchange of ligand pairs on the surface of NCs. This is never exactly a simple exchange procedure as the ligands themselves can be thought of as being part of the last layer of the NCs. Ligand replacement therefore involves a certain extent of etching. Understanding in more detail the ligand exchange processes requires a combination of several characterization techniques, such as optical and microscopy studies (for the careful evaluation of size/shape changes), elemental analyses (to highlight changes in the surface composition), and extended NMR spectroscopy to correctly identify the various surface-bound organic species. All of these measurements need to be coupled with accurate atomistic models that are able to correctly describe the outer NC layers. All of these studies can inform us on the limits to the surface tolerance of these NCs and address critical questions. For example, what is the critical density of surface vacancies above which NCs start to significantly lose their PLQY? What kinds of exogenous species (such as other inorganic cations/anions) can affect the surface and optical properties and stability of the NCs? All of these studies become particularly pressing as we move away from Cs-based perovskites and venture more into Pb-free metal halide NCs, in which surface tolerance is rarely observed.^30,40−46^

Our studies also highlight that a deep knowledge of the NC–ligand and ligand–solvent interactions is of the utmost importance in mastering the colloidal stability and optical properties of LHP NCs. In this regard, recently we proposed a thermodynamic model that combines the process of ligand binding/displacement at the NC surface and the process of precipitation/dissolution of NC–ligand complexes in organic solvents.^34^ We suggested that ideal ligands should simultaneously maximize the NCs’ surface coverage and dispersibility in a given solvent. Building upon these findings, in order to ensure good dispersibility, the ligands should prevent NCs’ aggregation, which can occur via interdigitation and via unbound regions of nearby NCs that can directly interact with each other. Nowadays, in most of the studies on LHP NCs not much attention is paid to the choice of ligands with a given anchor group and with different alkyl chains, as this would require the time-consuming empirical testing of numerous ligands (possible only through a large number of syntheses) before being able to identify a ligand that best matches the surface of an NC. Fortunately, the recent development of force-field parameters for classical molecular dynamics simulations enables the atomistic simulation of NC–ligand–solvent systems with reasonable NC sizes and ligands, and this can provide a comprehensive understanding of the dynamic surface region of colloidal NCs. We aim to use these simulation tools to investigate various parameters, such as ligand–ligand steric hindrance and ligand–core and ligand–solvent interactions to identify an optimal strategy to maximize surface coverage and improve both the colloidal stability and the optical properties of the NCs. However, the computational challenge of this type of study is remarkable, and it allows the exploration of only a restricted region of the ligands’ chemical space. Therefore, we believe that a more efficient approach to rationalizing and reducing the synthetic effort will be possible by integrating computational chemistry tools and machine learning models, similar to what is done in drug discovery. We believe that rapid computation of ligand properties that best describe ligands at the NC surface is a promising strategy to identify optimal ligand candidates in the future. Once a sufficient data set of ligand properties is generated, machine learning algorithms can be trained to predict high-throughput optimal ligand candidates that can be further assessed experimentally.
